# Advancing digital sensing in mental health research

**DOI:** 10.1038/s41746-024-01343-x

**Published:** 2024-12-18

**Authors:** Samir Akre, Darsol Seok, Christopher Douglas, Adrian Aguilera, Simona Carini, Jessilyn Dunn, Matthew Hotopf, David C. Mohr, Alex A. T. Bui, Nelson B. Freimer

**Affiliations:** 1 Medical Informatics Home Area, University of California Los Angeles, Los Angeles, CA USA; 2 Depression Grand Challenge, University of California Los Angeles, Los Angeles, CA USA; 3 Interdepartmental Program in Neuroscience, University of California Los Angeles, Los Angeles, CA USA; 4 Department of Psychiatry, School of Medicine, Washington University in St Louis, St Louis, MO USA; 5 School of Social Welfare, University of California Berkeley, Berkeley, CA USA; 6 Department of Psychiatry and Behavioral Sciences, Zuckerberg San Francisco General Hospital, University of California San Francisco, San Francisco, CA USA; 7 Department of Medicine, University of California San Francisco, San Francisco, CA USA; 8 Biomedical Engineering Department, Duke University, Durham, NC USA; 9 Department of Psychological Medicine, Institute of Psychiatry, Psychology and Neuroscience, King’s College London, London, United Kingdom; 10 Center for Behavioral Intervention Technologies, Department of Preventive Medicine, Northwestern University, Chicago, IL USA; 11 Medical & Imaging Informatics Group, University of California Los Angeles, Los Angeles, CA USA; 12 Center for Neurobehavioral Genetics, Semel Institute for Neuroscience and Human Behavior, David Geffen School of Medicine, University of California Los Angeles, Los Angeles, CA USA

**Keywords:** Human behaviour, Research management, Research data

## Abstract

Digital sensing tools, like smartphones and wearables, offer transformative potential for mental health research by enabling scalable, longitudinal data collection. Realizing this promise requires overcoming significant challenges including limited data standards, underpowered studies, and a disconnect between research aims and community needs. This report, based on the 2023 Workshop on Advancing Digital Sensing Tools for Mental Health, articulates strategies to address these challenges to ensure rigorous, equitable, and impactful research.

## Introduction

The data obtainable from the sensors on digital devices—including smartphones, smart watches, and other wearables – have the potential to transform research on mental health disorders by informing our understanding of their causes and enabling us to delineate underlying subtypes, predict their diverse trajectories, and identify potential points of intervention through observational studies and clinical trials. The field of mental health research, from its beginnings in the 19^th^ Century, has relied on subjective assessments, principally from self-report of symptoms and experiences, both to classify these disorders and to assess their course; these classifications are imprecise and encompass considerable heterogeneity^1–3^. More recently, researchers have had access to technologies such as neuroimaging and electroencephalography, which enable objective assessment of the relationships between emotions and behaviors and both physiologic and anatomic features. However, as these approaches have, to date, largely been limited to laboratory settings with limited ecological validity and have been difficult to scale, they have not yet demonstrated utility in providing more precise and less heterogeneous phenotypes for population-level mental health investigations.

Because consumer digital devices are now used pervasively and provide readouts on a wide range of behavioral and physiological variables, such as sleep, activity, and cardiovascular parameters like heart rate and heart rate variability, many researchers are optimistic that they will provide the first tools for objectively obtaining phenotypic assessments of mental health disorders at scale^4^. Such utility has not yet, however, been conclusively demonstrated. In this Perspective, we highlight key objectives that the field of digital mental health research must meet to fulfill its promise. We suggest here strategies for doing so, drawing on the recommendations of a 2023 international workshop, “Advancing the Utility of Digital Sensing Tools for Mental Health Research.”

Because mental health disorders are enormously heterogeneous, studies aiming to elucidate their etiology, define subgroups relevant to treatment choices, or predict their course require very large samples as effect sizes are inherently low. To accomplish any of these aims, digital sensing in mental health must decisively move beyond the exploratory studies that still constitute most of the literature in this field^5^. Well-powered discovery and replication studies will require researchers to increase sample sizes by orders of magnitude compared to current practice.

One way to increase scale will be to combine data across studies, leveraging the increasingly global ubiquity of consumer digital devices. However such a scale up will require concerted efforts to overcome several factors that act as impediments to data combination efforts, including the following: First, device manufacturers typically modify both hardware and software at frequent intervals and currently do so in ways that are largely opaque to investigators^6^. Further, as variables may be measured or reported in different ways, researchers must find ways to combine heterogeneous measurements. Additionally, researchers may be reluctant to share digital device data given uncertainties regarding the risks of such sharing^7^. And finally, effective data combination efforts will likely also depend on our obtaining a greater understanding of how socioeconomic variables and other group differences may influence the relationship between aspects of device usage and psychological or mental health measures^8^.

Overall, however, we consider the failure of the field to achieve agreed-upon standards for generating, interpreting, and sharing data to have played a crucial role in limiting its progress and believe that we can learn valuable lessons from other life science fields, such as human genomics, that have overcome similar challenges, as described below.

The release of the first draft of the human genome sequence in 2001 led quickly to coordinated efforts by the genomics field, supported by the US National Institutes of Health (NIH) and other funders to identify millions of genetic variants; the principal purpose of these efforts was to enable the discovery of genetic associations with common diseases, one of the central objectives of the Human Genome Project. While the availability of these variants led, over the ensuing few years, to a rapid growth in the number of genetic association studies performed for both common diseases and quantitative traits, these studies initially generated only a handful of replicated associations. Over this time, it became apparent that the lack of recognized standards for such studies was limiting the progress of the field, as noted in several methodological publications. By 2006, this awareness inspired two institutes at the US National Institutes of Health (the National Human Genome Research Institute [NHGRI] and the National Cancer Institute [NCI]) to jointly assemble a working group, including researchers, journal editors, and NHGRI/NCI staff, to propose guidelines that could constitute accepted standards for the design and reporting of genetic associations^9^. As a result of these and other efforts, the field quickly adopted rigorous criteria for considering associations statistically significant (both initial reports and replication efforts). These criteria, in turn, stimulated the design of studies that would be adequately powered to achieve such criteria, which required the field to rapidly develop a culture of extensive data sharing, effected both through the formation of international research consortia^10^ and the development of easily accessible data repositories^11^. Additionally, the need to analyze data across multiple studies hastened the spread of genotyping platforms that could inexpensively produce standardized data and the development and rapid acceptance of uniform analysis methods.

Taken together, the different steps that were followed to achieve standards for genetic association studies enabled a spectacular acceleration of progress, such that there are now millions of replicated associations for thousands of traits. These discoveries have had a tremendous impact across the biomedical sciences, from identification of biological mechanisms underlying diseases to clinical implementations for predictions of disease risk at the individual level^12^.

## 2023 Workshop on Advancing the Utility of Digital Sensing Tools for Mental Health Research

Taking a leaf from the experience of human genomics, thought leaders from multiple disciplines (drawn from academia, industry, funding and regulatory agencies, and groups representing individuals living with mental health disorders) have begun to grapple with the task of ensuring that mental health research using digital devices is scalable, yields reproducible findings, and can be conducted in ways that are both acceptable to diverse populations of research participants and useful to the communities from which they are drawn. Achieving this objective requires first delineating obstacles that impede progress and then identifying and implementing practical steps needed for the field to overcome these obstacles. The 53 participants in the 2023 digital sensing workshop, which was sponsored by the US National Institute of Mental Health (NIMH) and Wellcome, addressed both steps.

**Fig. 1:**
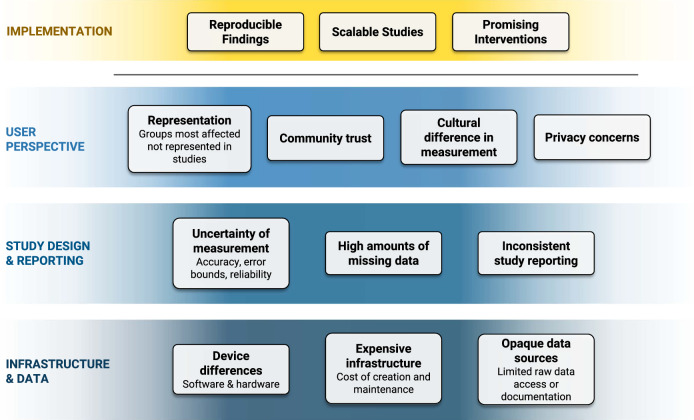
Overview of key issues discussed in the digital sensing workshop’s workgroups as they relate to key implementation goals for the field of behavioral digital sensing. The organizational structure shown here was adapted from a multilevel framework previously proposed by Mohr et al. for deriving and analyzing sensor features for mental health research^43^ The lowest level considers the sensors themselves (e.g., gyroscopes or accelerometers), on top of which are the low-level features derived from the sensors (e.g., types of activity or degrees of movement intensity), then higher-level behavioral markers (e.g., psychomotor activity or fatigue), and finally, clinical states (e.g., depression or anxiety). Figure 1 reframes the original figure in terms of key barriers to reaching goals in the field of behavioral digital sensing.

The workshop considered the obstacles to sensor-based assessments across three broad levels (Digital Infrastructure & Data Flow; Study Design & Reporting; and User Perspectives, Fig. 1), and its participants met in different workgroups that proposed solutions specific to each of these levels and produced reports that were made available for public comment (https://escholarship.org/uc/ucla_depression_grandchallenge). Below, we summarize the main challenges and recommendations identified by each group.

## Digital infrastructure and data flow

These two workgroups addressed how to effectively organize the wide range of sensors, apps, and data being used globally and how to harmonize pipelines and methods for collecting data from devices, analyzing results, and sharing key findings (Box 1).

Box 1 Digital infrastructure and data flow recommendations

1. Build standards for data from digital sensing devices
  - Address version changes and between-device differences for the same measure for hardware and software
  - Establish minimum requirements for a given measure (error bounds, sunset period, required metadata)
2. Fund Centers of Excellence centered on digital sensing for mental health to:
  - Create benchmark datasets
  - Maintain a network of sites with interdisciplinary expertise
  - Develop reusable assets for digital mental health studies starting with common APIs and endpoints
3. Identify the common ground between device manufacturers, academics, clinicians, and users
  - Define highest potential impact areas for sensor development
  - Establish protocols for ensuring privacy of participant data

### Device differences

Devices can be grouped by their intended use (clinical/research or consumer/wellness), although the lines between these uses are becoming more blurred every day^6,13,14^. The differences between devices, not only across categories but even different versions of the same device, have impeded efforts to replicate the results of digital behavioral sensing studies. As these differences are usually not transparent to most researchers, progress in the field depends on investigators gaining greater access to information on sensor components and specifications as well as the variables that are reported by the device. Differences between consumer platforms may be particularly important barriers to the equitable implementation of digital behavioral sensing, as the relative frequency of use of specific platforms varies considerably according to geographic and sociodemographic factors.

The building of standards for the types, formats, and parameters to adequately characterize data from consumer devices is one step toward being able to meaningfully compare data within and across studies. This process should leverage existing efforts, such as the IEEE Standard 1752.1-2021^15^, IEEE Working Group on digital health (P1752 Open Mobile Health)^16^, DiMe’s Digital Health Measurement Collaborative Community (DATAcc)^17^, and Open mHealth^18,19^. To facilitate further community and collaboration, it may be valuable to develop a network of expertise and an online community accessible to those conducting research on digital sensing in mental health (along the lines of programs such as Biostars for the bioinformatics community^20^) and to build on communities formed through the National Science Foundation (NSF) Smart and Connected Communities program^21^.

### Expensive infrastructure

Specifying standards is only a first step. The field will need accessible infrastructure to enable implementation of such standards in the process of large-scale data generation. Some current studies are collecting data on the scale of terabytes per participant over long time periods (in some cases several years), and the costs and complexity of this infrastructure are thus increasing rapidly. Efforts on this scale are not supportable with infrastructure that has largely developed on a bespoke, per-project basis, given rapid changes in technologies, application programming interfaces (APIs), and underlying data/communication standards. For this reason, scalable deployment of digital sensing in mental health research is now limited to a few sites with outsized resources and expertise. Even for such sites, the current landscape for funding does not adequately support the long-term maintenance of infrastructure or distribution of assets critical for research.

One mechanism for obtaining a more sustainable and equitable distribution of necessary infrastructure would be the formation of collaborative Centers of Excellence focused on digital sensing for mental health; establishing, supporting, and disseminating best practices in a sustainable manner; and curating shared datasets available to the scientific community. This strategy would incentivize groups to develop and maintain a set of reusable assets while pushing forward standards in the field. Key initiatives funded by this approach would be: 1) creating benchmark datasets; 2) maintaining a network of sites with interdisciplinary expertise; and 3) “priming the pump” for research through training and infrastructure buildout.

The creation of large benchmark datasets for digital sensing in mental health would simultaneously establish best practices that prioritize data privacy and sensitivity, while facilitating their dissemination to the research community. While mental health digital sensing data present distinct privacy concerns, other fields, such as genomics, have established standard practices for widespread sharing of similarly sensitive data. As device manufacturers may play a role in the generation of benchmark datasets, it will be important to engage these companies, from the outset of studies, in developing data sharing plans.

The interdisciplinary infrastructure of Centers of Excellence should initially focus on items that will remain scalable, such as common APIs and endpoints, and extend to include software for data aggregation and multimodal visualization (e.g., the Digital Biomarker Discovery Project^22^). Additionally, relying on postdoctoral scholars and graduate students for the maintenance of software infrastructure on this scale is not feasible. It will therefore be necessary for Centers of Excellence to include funding for research engineers over multiyear timespans. Importantly, this framework should reward investigators who enable reproducible research, prioritize underserved and underrepresented groups, and implement FAIR^23^ (Findable, Accessible, Interoperable, and Reusable) and TRUST^24^ (Transparency, Responsibility, User focus, Sustainability and Technology) principles.

### Opaque data sources

Device developers must navigate between their need for financially sustainable business models (e.g., proprietary and marketable technologies) and the demands from the research community for transparency in how their products function. The workgroup participants were emphatic in stating the view that fostering reproducible science does not require developers to reveal all their proprietary technology; indeed, it could strengthen their claims regarding the validity and efficacy of their devices. The participants further suggested that facilitating more extensive formal collaborations, throughout the process of developing and testing hardware and apps, could help bridge the gap between industry and academic sponsors. One of the workshop sponsors, the NIMH, has accepted this suggestion; in a recent announcement of a new funding opportunity, “Standardizing Data and Metadata from Wearable Devices, https://grants.nih.gov/grants/guide/pa-files/PAR-24-250.html, it strongly encouraged the inclusion of device manufacturers in research teams responding to this announcement.

To reach a state where we understand how specific device hardware and software versions influence measurements of interest (and what those measurements are) will require channels of communication between digital device companies, researchers, clinicians, funders, and users with lived experience. The continuation of workshops such as the one that generated this report and the leveraging of existing spaces where funding agencies, industry, and academic groups may already meet can foster such engagement. These discussions can provide three main “products” that will facilitate scalable research: 1) information on how software and hardware versioning and updates influence the sensor data that researchers gather; 2) well-documented APIs for gathering digital sensing data; and 3) creation of standards for what comprises data fit for research^25^. Additionally, as current consumer devices may not include some sensors that could have the greatest potential for impacting our understanding of mental health, efforts to foster iterative discussions between device developers and clinicians, academics, and those with lived experience could increase the chances that mental health applications will be considered when manufacturers make changes in the sensors on such devices.

## Study design and reporting

This workgroup sought to identify specific elements that are essential for successful digital sensing studies (as measured by significant and reproducible results). There is obviously no “one size fits all” formula; the design and reporting of exploratory studies will differ from hypothesis testing studies (e.g., those focused on identifying how digital sensing features map to specific existing psychological constructs), both of which will differ substantially from the design and reporting of clinical trials (e.g., evaluating whether the incorporation of digital sensing can improve specific clinical outcomes).

As noted in the Introduction, most published digital mental health sensing studies are vastly underpowered for either discovery or replication. There is not yet a sufficient body of literature reporting adequately powered digital sensing investigations that could serve as models in study design for other researchers. In the absence of such evidence, an important step that researchers could take to consider the appropriate scale of digital sensing studies that they are considering would be to, a priori, model as precisely as possible how digital sensing data types are hypothesized to relate to particular psychological or behavioral constructs, in different types of study samples. Values for these constructs will likely show more variability and change more rapidly in cohorts of individuals with diagnosed mental health disorders compared to population samples. Because of the lower expected effect sizes, researchers designing population studies must plan to investigate large samples for long durations. Additionally, as digital sensing studies generate data for a sizable number of distinct features, the need to correct statistical analyses for multiple testing can substantially affect their power; researchers should consider this factor in deciding on the frequency with which they assess the psychological or behavioral constructs that will be analyzed in relation to digital features.

The problem of statistical power is compounded by heterogeneity in multiple domains that can influence the relationships between sensor-derived features and mental health related targets, such as age, comorbid medical conditions, geographic location (e.g., urban vs. rural, latitude), culture, and socioeconomic status^8^. Additionally, while it is known that sociodemographic factors contribute substantially to the high degree of missing data characteristic of digital sensing studies, there is not yet a principled method to account for such missing data^26^.

Other medical informatics disciplines have demonstrated the value, for new fields, of establishing repositories for data sharing that can enable a wide community of researchers to apply a multitude of approaches to examine the relationships between measured variables and constructs of interest. An instructive example of such a data repository released for widespread analyses of sensitive medical data is that of the Medical Information Mart for Intensive Care (MIMIC) datasets, which required cross-sector collaboration to develop and have massively enabled innovation in computational medicine^27,28^ including novel imputation^29^ strategies.

Many research groups have acquired the experience and expertise to identify and adjust for common sources of errors when conducting these studies. Building from a review of digital health studies for the passive monitoring of depression by Angel et al.^5^, the workgroup assembled a list of recommendations for variables that researchers should consider when designing studies (Box 2). This list represents a current consensus of the workgroup participants for a *minimum* set of criteria and will evolve as the field matures.

Box 2 Recommendations for digital sensing study design and reporting **Study design and participant recruitment**

- Individuals with lived experience of mental health disorders should participate in the design of the study.
- Studies analyzing digital sensing data in relation to self-report instruments should collect information that will enable researchers to know when and in what situations participants are completing these instruments.
- Consider how contact between study staff and participants may impact study outcomes.
- Prioritize collection of data elements that can provide surrogate measures of data completeness or participant compliance with the study protocol. (e.g., hours of watch wear, phone off time)
- Mental health studies often include risk mitigation strategies for information gained from interviews or self-report assessments. Studies should consider what strategies may be warranted to mitigate the risk detected from digital sensing assessments.

**Study information**

Digital sensing studies should report:

- Setting, locations, and relevant dates, including periods of recruitment and data collection
- Processes used to promote participant adherence to the study protocol, including participant training, adherence monitoring, outreach processes, and participant compensation
- The sensor technology used, including make, model, versions, form factor, wear location, and sensor modalities (e.g., type, units, sampling rates, etc.)
- Missing data for all data types and techniques used to account for them
- Item-level responses in data from self-report instruments

Researchers should deposit data in access-controlled repositories such as PhysioNet or Sage Bionetworks.

**Data quality issues**

- Researchers should, if feasible, provide participants with the wearable devices from which study data are obtained as a means of controlling for device and sensor differences in software or hardware.
- The smartphones included in a study must be capable of collecting the sensor data of interest; therefore, researchers may consider the need to provide suitable phones and data plans to those who do not have them.
- Leverage existing digital infrastructure (data collection apps, data storage mechanisms) to avoid reinventing the wheel
- Routinely and continuously monitor data quality, including both missing data and outliers, to identify device and software failures or participants who are having difficulty adhering to protocols to allow for timely resolution of problems.

**Results**

- Assess algorithm and model performance stratified by demographic and clinical characteristics.
- Report limitations on how representative the dataset is to trained model generalizability

### Inconsistent reporting

In considering what steps would enable the field to trust reports of study findings, the group recognized that a lack of standardized formats for reporting results is a source of nonreproducibility of findings beyond those due to differences in platforms or to the issues of power discussed above. It is critical therefore that study reports address issues such as sufficiently characterizing the sensor technology, identifying the reference standard to which the technology is being compared, and documenting nonstandardized measures. As a step toward achieving standardization, the group proposed that reports should include standard checklists of methodology and outcomes and suggested the type of information that these checklists should include. One example of such a checklist is EVIDENCE-MH (EValuatIng connecteD sENsor teChnologiEs for Mental Health), available online at https://escholarship.org/uc/item/19c8w68w. This checklist adapts reporting frameworks developed in other areas of health science^30,31^ to the specific challenges of digital sensing in mental health. The checklist is not intended to duplicate existing guidelines for reporting research, such as CONSORT^32,33^ or STROBE^34^, but to specify aspects critical to digital sensing studies in mental health. By using EVIDENCE-MH, those preparing, reading, or reviewing personal sensing studies in mental health will be better equipped to determine whether a report has included all of the elements that are crucial for evaluating both the design of a study and its results.

## The users’ perspective

This workgroup explored ways to involve and engage potential users of digital sensing (people living with mental illness and their families or caregivers, mental healthcare providers, and representatives of diverse communities) in the design, implementation, and reporting of research based on these technologies.

### Representation, community trust, and cultural difference in measurement

Realizing the promise of digital sensing technologies in real-world settings requires the integration of different user perspectives from the outset. This point is especially true for research involving consumer devices, as the potential scale of impact encompasses entire populations. As such, it is imperative that researchers recognize the extent of historical and current inequities that have excluded minoritized communities and other marginalized populations from obtaining benefits from mental health research. These are the groups that are traditionally most impacted by mental health disorders, have had the least access to effective interventions for them, and have been underrepresented at all stages of research and development processes. This lack of representation can make research data biased and incomplete, which in itself reduces the generalizability of study findings. Research based on consumer devices faces a particular equity issue; if mental health digital sensing applications are developed predominantly for the most expensive devices, they may be inaccessible to large segments of the populations that need them. Thus, given the extent of current and historical inequities, ensuring inclusivity in digital health studies is imperative, for example, through the adoption of methodologies that we outline below^35^.

Digital health studies require distinct recruitment methodologies that reflect their reliance on personal devices with continuous tracking of individual behavior and physiology, often outside a traditional healthcare setting. To meaningfully engage diverse users, we propose user centered design and increased use of participatory action research (PAR), where community members are viewed as research partners who collaborate to define the problem, create solutions, test hypotheses, and provide iterative feedback. Toward this goal, we suggest the development of a resource to help mental health researchers using digital technologies to incorporate PAR. An outline of such a “Participatory Action Research Playbook” is shown in Box 3.

Incorporating PAR into digital sensing studies for mental health, for example by engaging Community Advisory Boards (CABs, including individuals with lived experience of mental health conditions) and cultural liaisons to interact with researchers from the initial stages of study design through the final stages of implementation, can greatly enhance the effectiveness of the research and its ultimate impact. For example, during the recruitment process a CAB can participate in community-focused meetings on privacy, which may increase the engagement of participants from historically marginalized communities. This PAR process guides the focus of a study towards designs that are best suited to such communities, for example by adopting protocols aimed at minimizing participant burden (a step that can improve protocol adherence and reduce the need for downstream troubleshooting).

While PAR approaches offer practical ways for acknowledging cultural differences in measurement, intersections of socioeconomic status with other identities, and aligning researcher and community needs, it may not be sufficient to foster trust with participants, especially in the context of events such as data breaches that have occurred at CrisisTextLine^36^, BetterHelp^37^, and other digital platforms. Two additional frameworks may be useful for this purpose the Digital Health Equity Framework (DHEF)^38^ and the Health Social Justice Guide (DHSJG)^39^. The DHEF provides a key resource to guide exploration of factors influencing health equity considering the multilevel influence of individual, interpersonal, community and societal factors. The DHSJG outlines key areas that should be considered in the development of digital health tools. Additional details on PAR and frameworks useful for enabling such research can be viewed in the working group report online at https://escholarship.org/uc/item/0p39q718.

Box 3 Participatory action research playbook overview **Benchmarking and literature review:** Conduct a thorough analysis of the target population, including a zip code analysis of needs, historical trauma, and cultural factors that affect the community's trust in research. This information will guide the research team in the development of the research question and hypothesis.

**Identifying cultural liaisons:** Identify and engage with cultural liaisons who can help the research team understand cultural factors that affect the community's perceptions of research. This will involve identifying individuals who have deep roots in the community and are trusted by community members.

**Creating an advisory board:** Create an advisory board that includes both internal and external partners. Internal partners should be individuals with authority who are committed to building trust and relevance, such as the Chief of Diversity, Equity, and Inclusion (DEI) within a leading organization. External partners should be community organizers or local NGOs who are already viewed as trusted parties.

**Community engagement and outreach strategies:** Develop and implement community engagement and outreach strategies that meet the community where they are. This process will involve going to where people congregate, such as schools or community centers, and using layperson's terms to communicate with clients and stakeholders. The research team should also consider providing culturally adapted translations of all materials.

**Prioritizing and aligning objectives:** Work with the community to identify common ground and set realistic expectations by articulating the mission statement and objectives of the research. This will help to manage expectations and mitigate unintended consequences and mission creep.

**Hypothesis and prototype development:** Use feedback from the community to develop a hypothesis and prototype that meet their needs and address the research question. This will involve creating upstream feedback loops and constantly engaging with the community to ensure the research stays on track.

**Testing and evaluation:** Test the hypothesis and prototype with the community and evaluate the effectiveness of the research. This will involve a constant feedback loop to ensure that the research remains relevant to the community's needs and concerns.

### Privacy concerns

A key focus area for future discussion is ethics and methods for privacy and deidentification of data. This is an area in which researchers require input from those in security-related fields, ethics experts, and those with lived experience to better understand what is required, what is possible, and how to build trusting partnerships. While people are willing to share their digital health data for research purposes^40–42^, constructing precise and protective informed consent is a challenge, given the granularity of data, the extent of data collection, the lack of control researchers have over third party technologies, and the potential for datasets to include people other than consented participants (e.g., in the case of voice or video recordings). The more complex the informed consent, the more complex its implementation, with the need to keep track of what data have been consented for sharing, for how long, with whom, and what effect the consent has on features derived from a given data type. We must accept that there is an inherent tradeoff, in consent forms, between interpretability and accessibility and a level of detail that encompasses all possible risks and ethical challenges. These challenges are especially salient in research conducted with populations that are unable to provide reliable subjective reports of their symptoms, based on their living with certain disorders or disabilities. Because digital sensing may offer unique possibilities for incorporating information about the experiences of such individuals within clinical care, it is imperative that they be included in research studies; the informed consent process must explicitly address privacy issues that are of particular concern to members of these populations.

## Conclusion

Research on digital sensing in mental health is a challenging domain, employing user-generated data to improve our understanding of the relationships between mental health disorders and a wide range of behavioral and physiologic variables. To enable reproducible science in the field will require the establishment of standards for obtaining, interpreting, and reporting sensor data, such as the use of the EVIDENCE-MH checklist. Increased support for research infrastructure, e.g., within new centers of excellence, will be essential for investigators to meet such standards. Finally, to develop equitably, the field of digital sensing in mental health must adopt a series of best practices to expand community engagement at all stages of research, for example, adoption of the Participatory Action Research Playbook as an initial baseline for study quality. It is only through concerted efforts in each of these areas that mental health research will achieve the groundbreaking advancements that digital sensing technology has made possible.

## Data Availability

No datasets were generated or analysed during the current study.
